# The survival rate of hepatocellular carcinoma in Asian countries: a systematic review and meta-analysis

**DOI:** 10.17179/excli2019-1842

**Published:** 2020-01-13

**Authors:** Soheil Hassanipour, Mouhebat Vali, Saber Gaffari-fam, Hossein-Ali Nikbakht, Elham Abdzadeh, Farahnaz Joukar, Akram Pourshams, Afshin Shafaghi, Mahdi Malakoutikhah, Morteza Arab-Zozani, Hamid Salehiniya, Fariborz Mansour-Ghanaei

**Affiliations:** 1 GI Cancer Screening and Prevention Research Center, Guilan University of Medical Sciences, Rasht, Iran; 2 Gastrointestinal and Liver Diseases Research Center, Guilan University of Medical Sciences, Rasht, Iran; 3 Student Research Committee, Shiraz University of Medical Sciences, Shiraz, Iran; 4 Road Traffic Injury Research Center, Tabriz University of Medical Sciences, Tabriz, Iran; 5 Social Determinants of Health Research Center, Health Research Institute, Babol University of Medical Sciences, Babol, Iran; 6 Caspian Digestive Disease Research Center, Guilan University of Medical Sciences, Rasht, Iran; 7 Digestive Oncology Research Center, Digestive Diseases Research Institute, Tehran University of Medical Sciences, Tehran, Iran; 8 Department of Occupational Health, Kashan University of Medical Sciences, Kashan, Iran; 9 Social Determinants of Health Research Center, Birjand University of Medical Sciences, Birjand, Iran; 10 Iranian Center of Excellence in Health Management, School of Management and Medical Informatics, Tabriz University of Medical Sciences, Tabriz, Iran

**Keywords:** survival rate, hepatocellular carcinoma, systematic review, meta-analysis, Asia

## Abstract

Hepatocellular carcinoma or Liver cancer (LC) is the sixth most common cancer and the fourth cause of death worldwide in 2018. There has not been a comprehensive study on the survival rate of patients with LC in Asia yet. Therefore, the present study was conducted to evaluate the survival rate of patients with LC in Asian countries. The methodology of the present study is based on the PRISMA (Preferred Reporting Items for Systematic Reviews and Meta-Analysis) statement. The researchers searched five international databases including Medline/PubMed, Scopus, Embase, Web of Knowledge and ProQuest until July 1, 2018. We also searched Google Scholar for detecting grey literature. The Newcastle-Ottawa Quality Assessment Form was used to evaluate the quality of selected papers. A total of 1425 titles were retrieved. 63 studies met the inclusion criteria. Based on the random-effect model one-year, three-year and five-year survival rate of LC were 34.8 % (95 % CI; 30.3-39.3), 19 % (95 % CI ; 18.2-21.8) and 18.1 % (95 % CI ;16.1-20.1) respectively. According to the results of our study, the LC survival rate in Asian countries is relatively lower than in Europe and North America.

## Introduction

Cancer is one of the major health problems today and is currently one of the leading causes of death in many countries (Bray et al., 2018[9]). Among malignancies, liver cancer (LC) is one of the most common one, of which more than 85 % of cases of LC has been observed in developing countries (Global Burden of Disease Cancer Collaboration et al., 2017[31]). The highest incidence of this cancer is in Asian and sub-Saharan Africa (Ladep et al., 2014[61]). Evidence suggests that the incidence of this cancer is soaring (McGlynn et al., 2015[77]), which has been observed in Europe, the Pacific, as well as Asian countries (La Vecchia et al., 2000[60]; Mirzaei et al., 2016[78]; Mohammadian et al., 2016[80]; Hassanipour et al., 2019[34]). In terms of mortality, the standardized mortality rate of LC is 5.9 per 100,000. Due to the very poor prognosis of LC, the mortality rate is 95 % (Torre et al., 2016[106]; Nikbakht et al., 2019[84]). Global variation in LC rates can be explained by the distribution of viral hepatitis B and C infection, that should be mentioned here, the two viruses alone account for 78 % of the total mortality rate of LC in the world (Zamor et al., 2017[122]; Jakupi et al., 2018[38]). There is also evidence that the incidence of LC in Asia is declining in many Asian countries as a result of hepatitis B vaccination (Mansour-Ghanaei et al., 2007[71]; Yeo et al., 2013[119]; Joukar et al., 2018[44]; Aniaku et al., 2019[5]). Liver cirrhosis is another major risk factor, of which 80-90 % of these patients have hepatocellular carcinoma (Gao et al., 2012[28]). LC is one of the malignancies that have been studied by genetics, and in this case the relative risk for a person with family history is 6.2 (Fernandez et al., 1994[27]; Turati et al., 2012[109]) and also it is significantly higher in males than in women (Naugler et al., 2007[83]).

 The main affecting factors for long-term survival in these patients are, early diagnosis of tumors, and treatment of patients with effective therapies (Wang et al., 2008[110]). Patients diagnosed at an early stage will be more likely to respond to treatment and have long-term survival (Yuen et al., 2000[120]) Also, in order to diagnose these patients at an early-stage, monitoring high risk people is a key and important factor (Zhang et al., 2004[125]). 

Survival rate is one of the most important health indicators that is essential in evaluating diagnostic and therapeutic programs. The first step to control the burden of disease related to cancers in any population, is to understand its status in the population, as well as to collect information about the incidence, survival, type and location of cancers. It is necessary to record effective indicators on the process and survival of cancers in a monitoring area and patients' information in order to perform the correct and appropriate treatment and to apply effective therapeutic methods and prevention strategies. In spite of increasing cancer treatment costs in developing countries, a few researches that were hospital-centered and population-based information have been conducted. Studies on the survival of LC in Asian countries have achieved various results, and the studied population in these surveys has also been different. Familiarity with the various years of survival rate of this cancer in different countries as well as different population groups can provide valuable information on the control, prevention, and treatment outcomes of patients with LC. There has not been a comprehensive study on the survival rate of patients with LC in Asia yet. Therefore, according to the mentioned points, the present study was conducted to evaluate the survival rate of patients with LC in Asian countries.

## Methods

The present study is a systematic review and meta-analysis study of LC survival rate in Asian countries. This study was designed and conducted in 2018. The methodology of the present study is based on the PRISMA (Preferred Reporting Items for Systematic Reviews and Meta-Analysis) statement (Moher et al., 2015[81]).

### Search strategy

The researchers searched five international databases including Medline/PubMed, Scopus, Embase, web of knowledge and ProQuest until July 1, 2018. We also searched the Google Scholar for detecting grey literature. Selected keywords for international databases included: (“neoplasm”, “cancer”, “carcinoma”, “malignancy”, “liver cancer”, “hepatocellular neoplasms”, “hepatocellular carcinoma”, “liver tumor”, “cancer of liver”, “neoplasms of liver”, “survival, “survival analysis”, “survival rate”, “Afghanistan”, “Armenia” ,“Azerbaijan”, “Bahrain” “Bangladesh”, “Bhutan”, “Brunei”, “Myanmar”, “Cambodia”, “China” “Georgia”, “Hong Kong”, “India”, “Indonesia”, “Iran”, “Iraq”, “Israel”, “Japan”, “Jordan”, “Kazakhstan” “North Korea”, “South Korea”, “Kuwait”, “Kyrgyzstan”, “Laos”, “Lebanon”, “Macau”, “Malaysia” “Maldives”, “Mongolia”, “Nepal”, “Oman”, “Pakistan”, ”Philippines”, “Qatar”, “Saudi Arabia”, “Singapore”, “Sri Lanka”, “Syria”, “Taiwan”, “Tajikistan”, “Thailand”, “Timor-Leste”, “Turkmenistan”, “United Arab Emirates”, “Uzbekistan” , ”Vietnam”, and “Yemen”). 

The initial search was conducted by two researchers (S.H and M.V). The searched record entered the EndNote X7 software, and duplicate articles were deleted. The search strategy of this study is presented in Supplementary Appendix 1.

### Inclusion and exclusion criteria

All observational studies (cross-sectional, case-control, and cohort) stated the survival rate of Localize LC in Asian countries were included in the study. Articles of other cancers reported survival in people who reported regional, metastatic, as well as review and meta-analysis studies were excluded. It should be noted that studies that did not report the sample size or confidence interval of survival rate were not included into the meta-analysis.

### Quality assessment

The Newcastle-Ottawa Quality Assessment Form was used to evaluate the quality of selected papers. This tool has 3 different parts including Selection (4 questions), Comparability (1 question) and Outcome (3 questions) and is based on the final scores divided into 3 categories Good (3 or 4 stars in selection domain and 1 or 2 stars in comparability domain and 2 or 3 stars in outcome/exposure domain), Fair (2 stars in selection domain and 1 or 2 stars in comparability domain and 2 or 3 stars in outcome/exposure domain) and Poor (0 or 1 star in selection domain or 0 stars in comparability domain or 0 or 1 stars in outcome/exposure domain) (Penson et al., 2012[89]). Result of quality assessment is presented in Suppplementary Appendix 2.

### Screening of studies

Screening of studies, extraction of results, and evaluation of quality control of articles were performed separately undependably by two authors (M.V and E.A). If there was no agreement between the two, the supervisor (F.M) would announce the final comment on that article.

### Data extraction form

All final articles entered into the study process were provided by a checklist that was previously prepared, and were arranged to extract the data. This checklist includes the name of the author, the year of publication, the period of the study, the country of origin, the survival rate by year for each survival period.

### Statistical analysis

The heterogeneity of the studies was assessed by Cochran test (with significance less than 0.1) and its composition using I^2^ statistics. In the case of heterogeneity, the random effects model was utilized with the inverse-variance method, and in the absence of heterogeneity, the fixed effects model was applied. In the case of a heterogeneity in the studies, methods such as subgroup analysis were used and factors like the geographical area and the HDI considered in the analysis of subgroups. All analyzes were performed by the STATA (version 13) software. 

### Additional analysis 

Due to the heterogeneity of the studies, the subgroups analysis was used. The indicator applied for this purpose is Human Development Indices and Indicators (HDI). The HDI is a relative measure of life expectancy, education, quality and education level, and in general, it is the living standards in human societies. This Index is estimated using the measure of welfare, especially among children and people of low age. These statistics can be used to measure the development of countries, the impact of economic policies on living standards, and the survival of LC in each of the countries was reported to provide a clear indication of the LC survival status in each country (Human Development Indices and Indicators, 2018[35]).

## Results

### Study selection

**Figure 1:**
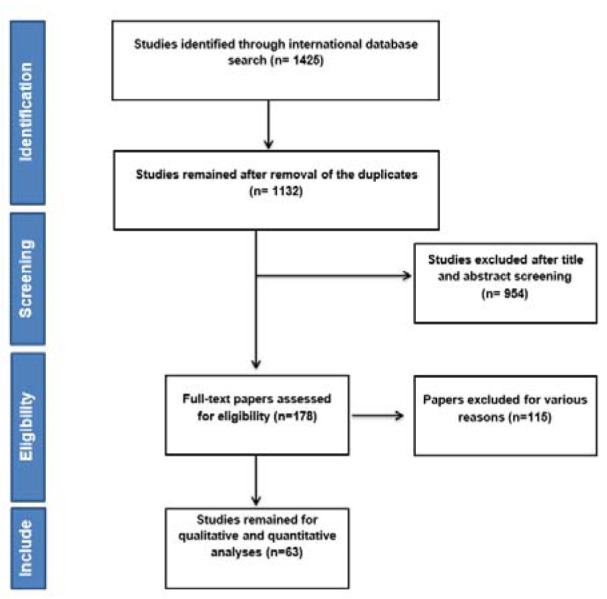
Flowchart of the included studies in systematic review

After searching the named international databases 1425 articles were selected and after removing duplicate articles 1132 remained. After reviewing the titles and abstract articles, the number of 178 articles entered the next stage, at which point the full text was examined and the 63 articles entered the final analysis. It should be noted that the referenced articles were also reviewed to add related studies. In the screening stages of studies, some articles were excluded for a variety of reasons, which included the unrelated topic (N=921), the unrelated population (N=133), inadequate information such as sample size, confidence interval and not reported overall survival in both sexes (N=12) and the repeated results (N=3). The study selection process is outlined in Figure 1(Fig. 1).

### Results of quality assessment

Based on our result, 30 studies had good quality and 33 studies had fair quality. The result of Quality Assessment is presented in Supplementary Appendix 1.

### Description of studies 

Based on the geographical location of 63 included studies (Sriamporn et al., 1995[99]; Chen et al., 1998[14]; Esteban et al., 1998[24]; Jin et al., 1998[43]; Martin et al., 1998[74]; Lee et al., 2000[64]; Chia et al., 2001[19]; Sato et al., 2002[95]; Toyoda et al., 2004[107]; Chen et al., 2006[12]; Tsukuma et al., 2006[108]; Yaghi et al., 2006[118]; Chen et al., 2007[16]; Jung et al., 2007[53]; Changchien et al., 2008[11]; Lim et al., 2009[68]; Redaniel et al., 2009[93]; Tanaka et al., 2009[104]; Laudico et al., 2010[62]; Chen et al., 2011[15]; Chia, 2011[18]; Jayalekshmi et al., 2011[40]; Jayant et al., 2011[41]; Jung et al., 2011[47]; Kudo et al., 2011[58]; Law and Mang, 2011[63]; Martin et al., 2011[73]; Matsuda et al., 2011[75]; Redaniel et al., 2011[92]; Sankaranarayanan, 2011[94]; Sriplung and Prechavittayakul, 2011[100]; Sumitsawan et al., 2011[101]; Xiang et al., 2011[114]; Xishan et al., 2011[116]; Azmawati and Krisnan, 2012[6]; Jung et al., 2012[48]; Chen et al., 2013[17]; Ito et al., 2013[37]; Jung et al., 2013[51]; Norsa'adah and Nurhazalini-Zayani, 2013[86]; Fan et al., 2014[26]; Ito et al., 2014[36]; Jung et al., 2014[46]; Pinheiro et al., 2014[90]; Somboon et al., 2014[98]; Jung et al., 2015[50]; Liu et al., 2015[69]; Maringe et al., 2015[72]; Xiao et al., 2015[115]; Zeng et al., 2015[124]; Zhang et al., 2015[126]; Zheng et al., 2015[129]; Zhu et al., 2015[131]; Chiang et al., 2016[20]; Kudo et al., 2016[59]; Oh et al., 2016[87]; Jung et al., 2017[52]; Li et al., 2017[66]; Nakagawa-Senda et al., 2017[82]; Chen et al., 2018[13]; Chien et al., 2018[21]; Jung et al., 2018[49]; Li-Hsin et al., 2018[67]), twenty-one studies were conducted in China, fourteen in Korea, nine in Japan, nine in Thailand, nine in Taiwan, four in Philippines, four in Singapore, four in India, two in Malaysia, one in Hong-Kong and one study in south Asian countries. The summary characteristics of the included studies were shown in Table 1(Tab. 1) (References in Table 1: Azmawati, 2012[6]; Changchien, 2008[11]; Chen, 1998[14]; Chen, 2006[12]; Chen, 2007[16]; Chen, 2011[15]; Chen, 2013[17]; Chen, 2018[13]; Chia, 2001[19]; Chia, 2011[18]; Chiang, 2016[20]; Chien, 2018[21]; Esteban, 1998[24]; Fan, 2014[26]; Ito, 2013[37]; Ito, 2014[36]; Jayalekshmi, 2011[40]; Jayant, 2011[41]; Jin, 1998[43]; Jung, 2007[53]; Jung, 2011[47]; Jung, 2012[48]; Jung, 2013[51]; Jung, 2014[46]; Jung, 2015[50]; Jung, 2017[52]; Jung, 2018[49]; Kudo, 2011[58]; Kudo, 2016[59]; Laudico, 2010[62]; Law, 2011[63]; Lee, 2000[64]; Li, 2017[66]; Li-Hsin, 2018[67]; Lim, 2009[68]; Liu, 2015[69]; Maringe, 2015[72]; Martin, 1998[74]; Martin, 2011[73]; Matsuda, 2011[75]; Nakagawa-Senda, 2017[82]; Norsa'adah, 2013[86]; Oh, 2016[87]; Pinheiro, 2014[90]; Redaniel, 2009[93]; Redaniel, 2011[92]; Sankaranarayanan, 2011[94]; Sato, 2002[95]; Somboon, 2014[98]; Sriamporn, 1995[99]; Sriplung, 2011[100]; Sumitsawan, 2011[101]; Tanaka, 2009[104]; Toyoda, 2004[107]; Tsukuma, 2006[108]; Xiang, 2011[114]; Xiao, 2015[115]; Xishan, 2011[116]; Yaghi, 2006[118]; Zeng, 2015[124]; Zhang, 2015[126]; Zheng, 2015[129]; Zhu, 2015[131]).

### Heterogeneity 

The result of chi-squared test and the I^2^ index indicated that there was a considerable between-study heterogeneity. For one (I^2^= 99.8 %, P<0.001), three (I^2^= 99.8 %, P<0.001), five (I^2^= 99.9 %, P<0.001) and ten- year survival rate (I^2^= 99.6 %, P<0.001).

### Synthesis of results

The articles were sorted according to the year of publication, and then analyzed by survival analysis of 1, 3, 5 and 10 years survival rate based on random effect model.

### One-year survival rate

**Figure 2:**
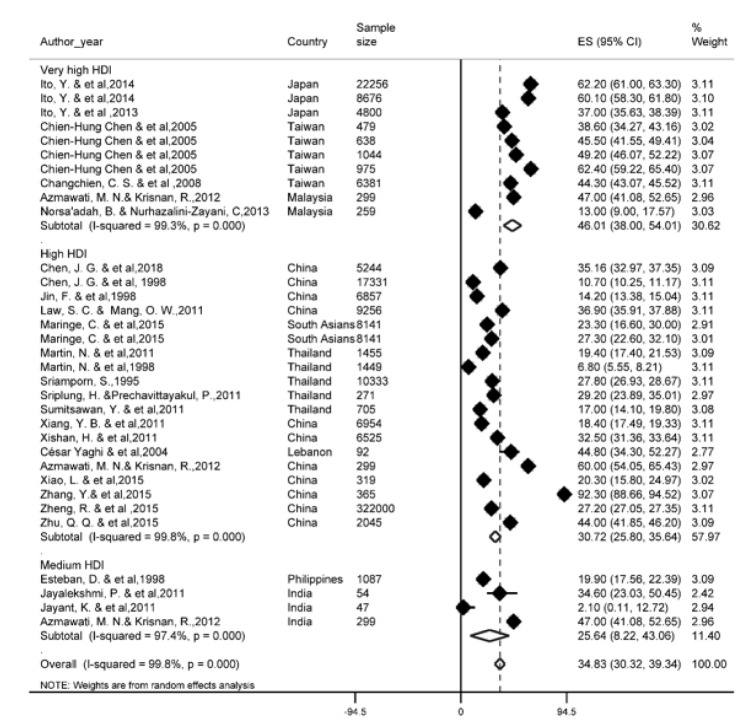
Forest plot of one-year survival rate of liver cancer in Asian countries

Of the most recent papers, 27 studies reported a one-year survival rate. The results of the study showed that one-year survival rate in Asian countries was 34.8 % (95 % CI; 30.3- 39.3). One-year survival rate of LC based on HDI has been shown in Figure 2(Fig. 2) (References in Figure 2: Azmawati, 2012[6]; Changchien, 2008[11]; Chen, 1998[14]; Chen, 2018[13]; Esteban, 1998[24]; Ito, 2013[37]; Ito, 2014[36]; Jayalekshmi, 2011[40]; Jayant, 2011[41]; Jin, 1998[43]; Law, 2011[63]; Maringe, 2015[72]; Martin, 1998[74]; Martin, 2011[73]; Norsa'adah, 2013[86]; Sriamporn, 1995[99]; Sriplung, 2011[100]; Sumitsawan, 2011[101]; Xiang, 2011[114]; Xiao, 2015[115]; Xishan, 2011[116]; Zhang, 2015[126]; Zheng, 2015[129]; Zhu, 2015[131]). Regarding the results, the highest one-year survival rate in countries with a very high HDI level (46 %, 95 % CI; 38-54) and the lowest was observed among countries with medium HDI level (25.6 %, 95 % CI; 8.2-43.6). 

### Three-year survival rate

**Figure 3:**
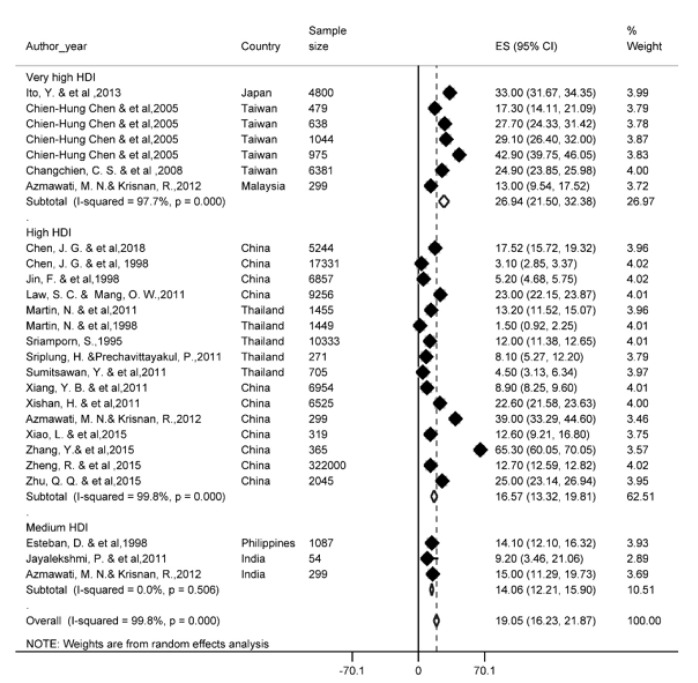
Forest plot of three-year survival rate of liver cancer in Asian countries

There were 22 studies that reported 3-year survival rate. Based on the results of our study, the 3-year survival rate in Asian countries was 19 %, (95 % CI; 16.2-21.8). The three-year survival rate of LC based on HDI is presented in Figure 3(Fig. 3) (References in Figure 3: Azmawati, 2012[6]; Changchien, 2008[11]; Chen, 1998[14]; Chen, 2018[13]; Esteban, 1998[24]; Ito, 2013[37]; Jayalekshmi, 2011[40]; Jin, 1998[43]; Law, 2011[63]; Martin, 1998[74]; Martin, 2011[73]; Sriamporn, 1995[99]; Sriplung, 2011[100]; Sumitsawan, 2011[101]; Xiang, 2011[114]; Xiao, 2015[115]; Xishan, 2011[116]; Zhang, 2015[126]; Zheng, 2015[129]; Zhu, 2015[131]). According to the results, the highest three-year survival rate for countries with a high HDI level (26.9 %, 95 % CI; 32.3-21.5) and the lowest for countries with the medium HDI levels was 14 % (95 % CI; 18.2-12.29). 

### Five-year survival rate

**Figure 4:**
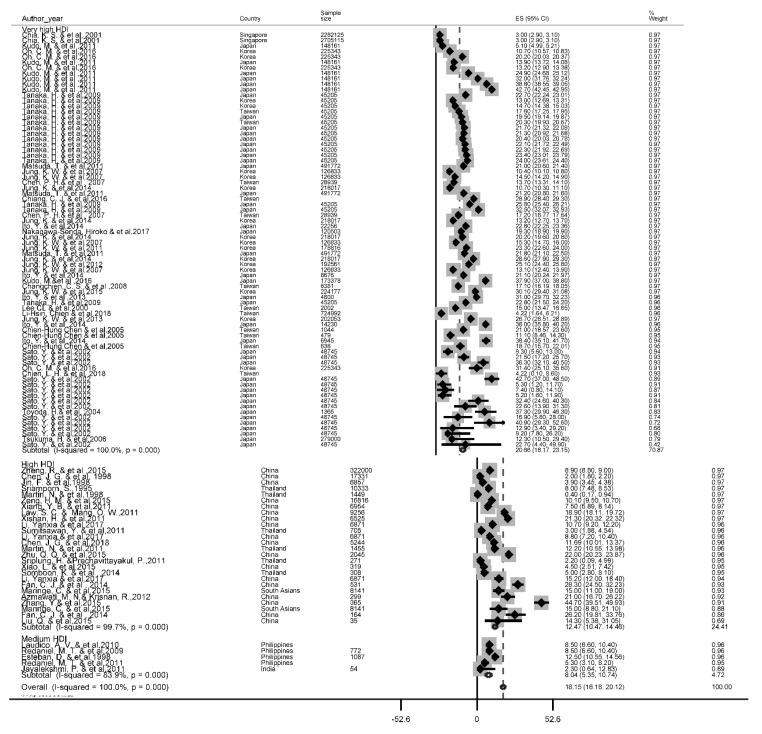
Forest plot of five-year survival rate of liver cancer in Asian countries

A total of 56 studies reported this survival rate. The 5 years rate of LC was 18.1 % (95 % CI, 16.2-20.1). The results of the 5-year survival by the HDI is depicted in Figure 4(Fig. 4) (References in Figure 4: Azmawati, 2012[6]; Changchien, 2008[11]; Chen, 1998[14]; Chen, 2007[16]; Chen, 2018[13]; Chia, 2001[18]; Chiang, 2016[20]; Chien, 2018[21]; Esteban, 1998[24]; Fan, 2014[26]; Ito, 2013[37]; Ito, 2014[36]; Jayalekshmi, 2011[40]; Jin, 1998[43]; Jung, 2007[53]; Jung, 2011[47]; Jung, 2012[48]; Jung, 2013[51]; Jung, 2014[46]; Jung, 2015[50]; Kudo, 2011[58]; Laudico, 2010[62]; Law, 2011[63]; Lee, 2000[64]; Li, 2017[66]; Li-Hsin, 2018[67]; Liu, 2015[69]; Maringe, 2015[72]; Martin, 1998[74]; Matsuda, 2011[75]; Nakagawa-Senda, 2017[82]; Oh, 2016[87]; Redaniel, 2009[93]; Redaniel, 2011[92]; Sato, 2002[95]; Somboon, 2014[98]; Sriamporn, 1995[99]; Sriplung, 2011[100]; Sumitsawan, 2011[101]; Tanaka, 2009[104]; Toyoda, 2004[107]; Tsukuma, 2006[108]; Xiang, 2011[114]; Xiao, 2015[115]; Xishan, 2011[116]; Zeng, 2015[124]; Zhang, 2015[126]; Zheng, 2015[129]; Zhu, 2015[131]). Based on the findings of our study, the highest five-year survival rate for countries with very high HDI levels (20.7 %, 95 % CI; 18.2-23.2) and the lowest was for the countries with the medium HDI level 8 %, 95 % CI; 5.3-10.7). 

### Ten-year survival rate

**Figure 5:**
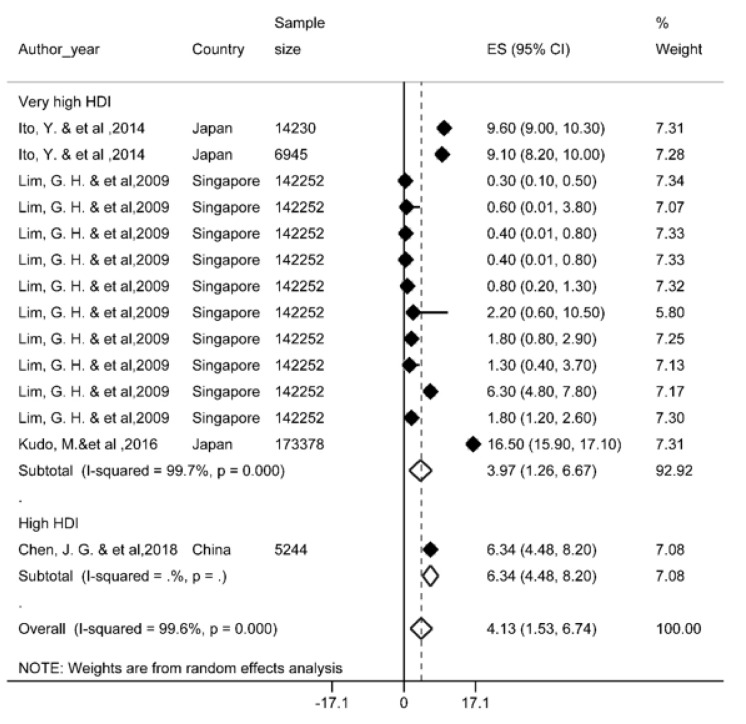
Forest plot of ten-year survival rate of liver cancer in Asian countries

Four studies reported this survival rate and based on the results, the ten-year survival rate was 4.1 % (95 % CI; 1.5-6.7). Ten-year survival of LC by HDI has been shown in Figure 5(Fig. 5) (References in Figure 5: Ito, 2014[36]; Lim, 2009[68]; Kudo, 2016[59]; Chen, 2018[13]). Given the limited number of studies conducted for 10-year survival rate, data on countries with medium HDI levels was not available. 

## Analysis of Subgroups

### Survival rate of liver cancer in each country

Overall, the results of the survival of the LC in ten countries and another region has been reported in Table 2(Tab. 2). The highest survival rates of one, three, five, and ten years were reported in Japan and the lowest survival rates for these years were observed in countries such as the Philippines, Thailand, India and Singapore.

### Meta-regression

Results of meta-regression showed a significant association between publication year and survival rate. Thus, year of study is a cause of variability in results of one (Reg Coef= 0.041, *p*=0.002), three (Reg Coef= 0.049, *p*=0.017) and five year survival rate (Reg Coef= 0.036, *p*<0.001). According to results, an increasing survival rate across the study period was observed. Results of meta-regression has been shown in Supplementary Appendix 3.

## Discussion

LC is the sixth most common cancer and the fourth cause of death worldwide in 2018, with 841,000 new cases and 782,000 deaths annually (Bray et al., 2018[9]). The incidence and mortality of men are two to three times higher than women in most parts of the world (Altekruse et al., 2014[3]). 

The results of our study showed that one, three, five and ten-year survival rate of LC in Asian countries was 34.8 %, 19 %, 18.1 %, and 4.1 % respectively. Significant heterogeneity was seen in the between studies. The present study was conducted in identifying high-risk individuals, diagnosis and early treatment of LC. The most important treatment for hepatocellular carcinoma is hepatic resection, trans-arterial embolization, percutaneous ethanol injection therapy, regional chemotherapy, and liver transplantation (Takano et al., 2000[102]; Fakhar et al., 2016[25]; Zhao et al., 2019[127]). For instance, 84.5 % of patients in Pakistan (Yusuf et al., 2007[121]) did not receive any treatment interventions, which was 30.5 % in China (Zhou et al., 2000[130]). Mortality rate for all cancers are 50 % more common in men than in women. Mortality rate in men were 171 per 100,000 in East Africa to 67.4 in Central America, ranging from 7.1 in Melanesia to 2.6 in Central and East Asia (except China) (Ghoncheh and Salehiniya, 2016[30]). The cumulative mortality risk from cancer among women in East Africa (11.4 %) in 2018 was higher than the estimated risk in North Africa (8.6 %), Northern Europe (9.1 %), and Australia / New Zealand (1.8 %) (Allemani et al., 2018[2]; Bray et al., 2018[9]). In one study which is conducted on localized staged patients treated with invasive methods, blacks have a 12 percent higher mortality rate, while Asian or Pacific islanders have a 16 percent lower mortality rate compared to whites (Wong and Corley, 2009[112]).

The statistics show that about half of the cases of death from primary LC occurred in China (Zheng et al., 2018[128]). The variety of survival rates in Asian countries includes the range of medical and pharmaceutical care and coverage of insurance services, socioeconomic status, ethnicity and lifestyle (Williams et al., 2010[111]). Medical advances have been made in detecting small tumors with a variety of scanning techniques (Takayasu et al., 1995[103]). 

In our analysis, the one-year survival rate in Asian countries was 34.8 %. The one-year survival rate is related to factors such as age older than 50 years, CLIP score <3, ALP <120 U/l, LDH <450 IU/l, CRP <0.8 mg/dl, tumor size less than 6 cm, disease stage, and Child-Pugh less than 7 that is associated with increased mortality (Toyoda et al., 2004[107]; Georgiades et al., 2006[29]; Jun et al., 2013[45]; Agarwal et al., 2015[1]). A meta-analysis study concluded that Transarterial chemoembolization (TACE) in patients with portal vein thrombosis significantly increased the 6 months survival rate (Xue et al., 2013[117]). However, haptoglobin (Hp) serum could be a potential and alternative contributor to alpha-fetoprotein. Contradiction in predicting one year rate in various geographic regions can be due to the selecting patients with demographic and clinical characteristics, diagnostic criteria, severity of the cirrhosis, number and size of the tumor (Shu et al., 2010[97]; Moayedi et al., 2019[79]). Despite the limited number of HCTs available for HCC, one-year survival rates for HCC have almost doubled over the period of 1992-1993 to 2003-2004 in the United States (Altekruse et al., 2009[4]). Of course, this increase in survival is limited to short-term follow-up. In addition, with aggressive treatments including liver transplantation and resection for localized-stage tumors, they are effective in boosting survival rates (Schwarz and Smith, 2008[96]). It is also associated with the expansion of public health and oncology care (Bai et al., 2018[7]).

Based on the results, the five-year survival rate was 18.1 %. The 5-year survival rate of all cancers in Korea in each sex, reached to 7.70 % (2011-2015) from 2.41 % (1995-1993) (Jung et al., 2018[49]). Besides, the five-year survival rate of LC in Shanghai in 1990 was 0.9 percent, reaching 0.1 percent in the last decade (Xiang et al., 2011[114]; Han et al., 2012[32]). Uninodular tumors and non-vascular invasion were associated with a 5-year survival (Jeong et al., 2017[42]). Liver transplant increases 5-year survival to 75 %, which is the best treatment option (Zamora-Valdes et al., 2017[123]). A meta-analysis study of 19 researches illustrated that the median survival rate for 436 patients with liver resection for hepatocyte metastasis to gastric was 17 months and a 5-year survival of 26.5 % (Kerkar et al., 2010[54]). An overall improvement in community health and the use of anti-viral therapies is effective in extending patient survival. Liver transplantation is an appropriate option (Omata et al., 2010[88]; Zhu et al., 2015[131]). However, the high cost of transplantation and the low number of donors of liver transplantation in most cases make it impractical (Llovet et al., 2005[70]). Resection surgery is recommended as a treatment for BCLC (Barcelona Clinic Liver Cancer) in very early and primary phases without portal hypertension and abnormal bilirubin. However, resection surgery is now the first line of therapy and leads to better patient survival (Dimitroulis et al., 2017[22]). 

The gender variable is an independent predictor in the prognostic primary liver cancer (PLC), regardless of the death of the PLC and all causes of death (Chen et al., 2007[16]). However, in other studies, the gender variable was not a predictor of survival due to PLC (Tangkijvanich et al., 2004[105]; Jarnagin et al., 2009[39]). Previous studies have shown that women have a higher rate of survival after surgery. Differences in aspects of medical interventions are attributed to gender disparity in survival rates (Wu et al., 2018[113]). The high alcohol intake in men increases cirrhosis of the liver towards the PLC (Nordenstedt et al., 2010[85]; Kröner et al., 2015[57]). 

Analysis of subgroups based on the geographic region show that, the highest survival rates of one, three, five, and ten years are reported in Japan and the lowest survival rates for these years were observed in the Philippines, Thailand, India and Singapore, respectively. The only areas with a high prevalence rate in the past include Japan, Korea and China, which have witnessed a sharp decline in outbreaks in recent years (Bertuccio et al., 2017[8]).

According to the latest global release, the highest incidence has taken place in lower HDI settings, and LC in 13 countries was one of the most common malignancies, including several countries in North and West Africa (Egypt, Gambia, Guinea) and the East and South East Asia (Mongolia, Cambodia and Vietnam) (Hashim et al., 2016[33]). Also, over the most recent years, the mortality rate of HCC is 2 to 5 times higher in Japan, Hong Kong and Korea than in European and American countries (McGlynn and London, 2011[76]; Zhu et al., 2016[132]). In the case of China, the proportion of Child A was less prevalent, while this proportion was more prevalent in the case of the Italian Child B type (Pons et al., 2005[91]; Azmawati and Krisnan, 2012[6]; Kew, 2014[55]). In addition, Asians have lower survival rates than non-Asians (Chang et al., 2007[10]). Advances have been made in HCC management and treatment, through local ablation, hepatic resection and liver transplantation. The impact of such improvements on HCC mortality was limited, as its 5-year survival rate was about 10 % (Lepage et al., 2015[65]). The five-year survival rate of the cancer in the US rose from 3 % over the 1975-1977 period to 11 % in the period of 2001-2007. While this survival rate ranged from 10.7 % in 1975-1977 to 18.9 % in the period 2001-2005. Approximately 62 % of HCC patients in Japan have undergone resection or ablation as primary care (Korean Liver Cancer Study Group and National Cancer Center Korea, 2015[56]). By comparing these proportions, only 30 % of these patients in the Western countries by the initial diagnosis were under these treatment interventions. Perhaps the care of high-risk patients will cause early detection and early diagnosis in countries such as Japan and Korea (El-Serag and Davila, 2011[23]).

### Study limitations

There are certain limitations in systematic review studies, most notably in the absence of access to some of the information that attempts were made to contact the authors of the study to resolve the problem, which in several cases did not receive an adequate response. One of the main limitation in our study was the failure to report sample size and the inability to calculate the confidence interval for survival, which did not allow the study to include the meta-analysis stage. Other limitations included a survival report of less than one year (6 and 9 months), which, given the low level of these, had no significant effect on our results. Ultimately, due to the lack of studies reporting 10-year survival, the correct estimate of survival requires more robust studies.

### Recommendations for future research 

According to the results of this study, estimating the survival rate of LC requires more extensive studies at the level of other Asian countries, especially in the West and Central Asia, as most studies in this study were conducted in South and Southeast of Asia, and estimates are somewhat incorrect. Another suggestion could be a study of the survival of LC in patients who metastasized, which was not our study goal, and is an important issue in clinical decision-making and the continuation of treatment.

## Conclusion

According to the results of our study, the LC survival rate in Asian countries is relatively lower than in Europe and North America, which may be due to less access to diagnostic facilities and higher age at recognition of disease than to advanced countries. Another result of our study was the higher survival rate of LC in countries with very high HDI (such as South Korea and Japan), with similar survival rates within advanced countries such as Europe and North America.

## Author contributions

Conceived and designed the experiments: S.H and F.M

Collected the data: M.V, H.N and E.A

Analyzed the data: S.H, F.J, M.A and H.S.

Wrote the paper: S.G, H.N, A.P and A.S. 

Revised the paper: S.H, F.M and F.J.

## Funding

This study was supported financially by Guilan University of Medical Sciences, Rasht, Iran.

## Conflict of interests

The authors have no conflict of interest to disclose.

## Supplementary Material

Supplementary material

## Figures and Tables

**Table 1:**
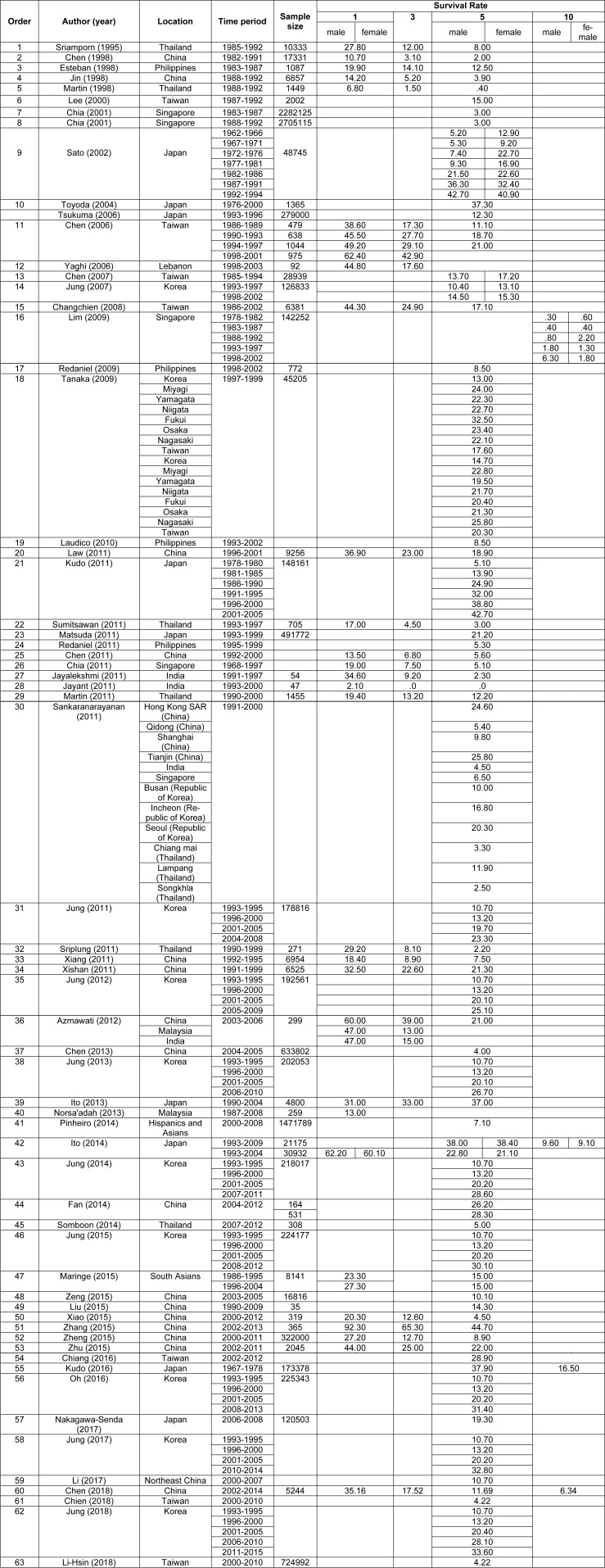
Basic information of included studies

**Table 2:**
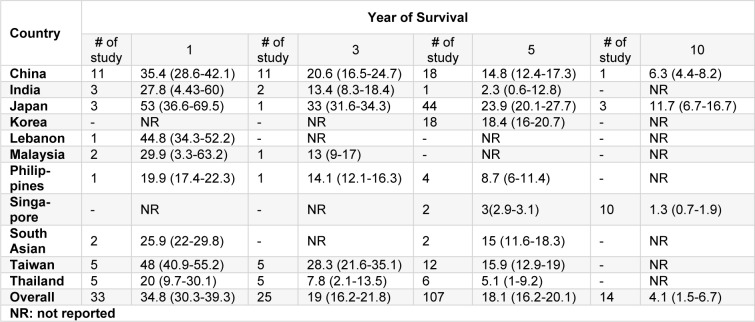
Result of meta-analysis of survival rate of liver cancer in Asia based on each country and year of survival
