# Supplementary material for: The survival rate of hepatocellular carcinoma in Asian countries: a systematic review and meta-analysis
Source: EXCLI J. 2020 Jan 13;19:108–30. doi: 10.17179/excli2019-1842 (PMC7003639; doi:10.17179/excli2019-1842)

EXCLI Journal 2020;19:108-130 – ISSN 1611-2156
Received: September 23, 2019, accepted: November 19, 2019, published: January 13, 2020

Supplementary material to:

THE SURVIVAL RATE OF HEPATOCELLULAR CARCINOMA IN ASIAN COUNTRIES: A SYSTEMATIC REVIEW AND META-ANALYSIS

Soheil Hassanipour[1, 2], Mouhebat Vali[3], Saber Gaffari-fam[4], Hossein-Ali Nikbakht[5], Elham Abdzadeh[1], Farahnaz Joukar[2, 6], Akram Pourshams[2, 7], Afshin Shafaghi[6], Mahdi Malakoutikhah[8], Morteza Arab-Zozani[9, 10], Hamid Salehiniya[9], Fariborz Mansour-Ghanaei[1, 2, 6*]

1 GI Cancer Screening and Prevention Research Center, Guilan University of Medical Sciences, Rasht, Iran
2 Gastrointestinal and Liver Diseases Research Center, Guilan University of Medical Sciences, Rasht, Iran
3 Student Research Committee, Shiraz University of Medical Sciences, Shiraz, Iran
4 Road Traffic Injury Research Center, Tabriz University of Medical Sciences, Tabriz, Iran
5 Social Determinants of Health Research Center, Health Research Institute, Babol University of Medical Sciences, Babol, Iran
6 Caspian Digestive Disease Research Center, Guilan University of Medical Sciences, Rasht, Iran
7 Digestive Oncology Research Center, Digestive Diseases Research Institute, Tehran University of Medical Sciences, Tehran, Iran
8 Department of Occupational Health, Kashan University of Medical Sciences, Kashan, Iran
9 Social Determinants of Health Research Center, Birjand University of Medical Sciences, Birjand, Iran
10 Iranian Center of Excellence in Health Management, School of Management and Medical Informatics, Tabriz University of Medical Sciences, Tabriz, Iran

* **Corresponding author:** Fariborz Mansour-Ghanaei, Gastrointestinal and Liver Diseases Research Center, Guilan University of Medical Sciences, Razi Hospital, Sardar-Jangle Ave., P.O. Box: 41448-95655, Rasht, Iran. Tel: +98(13)33535116, Fax: +98(13)33534951, E-mail:fmansourghanaei@gmail.com

http://dx.doi.org/10.17179/excli2019-1842

This is an Open Access article distributed under the terms of the Creative Commons Attribution License (http://creativecommons.org/licenses/by/4.0/).

EXCLI Journal 2020;19:108-130 – ISSN 1611-2156
Received: September 23, 2019, accepted: November 19, 2019, published: January 13, 2020

Supplementary Appendix 1: Search strategy in PubMed

#1 "Liver Neoplasms"[Mesh]

#2 ("Survival Rate"[Mesh]) OR "Survival Analysis"[Mesh]

#3 ("Asia"[Mesh]) OR Afghanistan[Title/Abstract]) OR Bahrain[Title/Abstract]) OR Bangladesh[Title/Abstract]) OR Bhutan[Title/Abstract]) OR Brunei[Title/Abstract]) OR Myanmar[Title/Abstract]) OR Cambodia[Title/Abstract]) OR China[Title/Abstract]) OR Hong Kong[Title/Abstract]) OR India[Title/Abstract]) OR Indonesia[Title/Abstract]) OR Iran[Title/Abstract]) OR Iraq[Title/Abstract]) OR Japan[Title/Abstract]) OR Jordan[Title/Abstract]) OR Kazakhstan[Title/Abstract]) OR North Korea[Title/Abstract]) OR South Korea[Title/Abstract]) OR Korea[Title/Abstract]) OR Kuwait[Title/Abstract]) OR Kyrgyzstan[Title/Abstract]) OR Laos[Title/Abstract]) OR Lebanon[Title/Abstract]) OR Macau[Title/Abstract]) OR Malaysia[Title/Abstract]) OR Maldives[Title/Abstract]) OR Mongolia[Title/Abstract]) OR Nepal[Title/Abstract]) OR Oman[Title/Abstract]) OR Pakistan[Title/Abstract]) OR Philippines[Title/Abstract]) OR Qatar[Title/Abstract]) OR Saudi Arabia[Title/Abstract]) OR Singapore[Title/Abstract]) OR Sri Lanka[Title/Abstract]) OR Syria[Title/Abstract]) OR Taiwan[Title/Abstract]) OR Chinese Taipei[Title/Abstract]) OR Tajikistan[Title/Abstract]) OR Thailand[Title/Abstract]) OR Timor-Leste[Title/Abstract]) OR Turkmenistan[Title/Abstract]) OR Uzbekistan[Title/Abstract]) OR Vietnam[Title/Abstract]) OR Yemen [Title/Abstract])

#4 (("Liver Neoplasms"[Mesh]) AND (("Survival Rate"[Mesh]) OR "Survival Analysis"[Mesh])) AND ((("Asia"[Mesh]) OR Afghanistan[Title/Abstract]) OR Bahrain[Title/Abstract]) OR Bangladesh[Title/Abstract]) OR Bhutan[Title/Abstract]) OR Brunei[Title/Abstract]) OR Myanmar[Title/Abstract]) OR Cambodia[Title/Abstract]) OR China[Title/Abstract]) OR Hong Kong[Title/Abstract]) OR India[Title/Abstract]) OR Indonesia[Title/Abstract]) OR Iran[Title/Abstract]) OR Iraq[Title/Abstract]) OR Japan[Title/Abstract]) OR Jordan[Title/Abstract]) OR Kazakhstan[Title/Abstract]) OR North Korea[Title/Abstract]) OR South Korea[Title/Abstract]) OR Korea[Title/Abstract]) OR Kuwait[Title/Abstract]) OR Kyrgyzstan[Title/Abstract]) OR Laos[Title/Abstract]) OR Lebanon[Title/Abstract]) OR Macau[Title/Abstract]) OR Malaysia[Title/Abstract]) OR Maldives[Title/Abstract]) OR Mongolia[Title/Abstract]) OR Nepal[Title/Abstract]) OR Oman[Title/Abstract]) OR Pakistan[Title/Abstract]) OR Philippines[Title/Abstract]) OR Qatar[Title/Abstract]) OR Saudi Arabia[Title/Abstract]) OR Singapore[Title/Abstract]) OR Sri Lanka[Title/Abstract]) OR Syria[Title/Abstract]) OR Taiwan[Title/Abstract]) OR Chinese Taipei[Title/Abstract]) OR Tajikistan[Title/Abstract]) OR Thailand[Title/Abstract]) OR Timor-Leste[Title/Abstract]) OR Turkmenistan[Title/Abstract]) OR Uzbekistan[Title/Abstract]) OR Vietnam[Title/Abstract]) OR Yemen[Title/Abstract])).

EXCLI Journal 2020;19:108-130 – ISSN 1611-2156
Received: September 23, 2019, accepted: November 19, 2019, published: January 13, 2020

Supplementary Appendix 2: Result of Newcastle-Ottawa Quality Assessment Form

Author (year)	Selection	Comparability	Outcome	Total	Quality
Sriamporn (1995)	2	1	2	5	Fair
Chen (1998)	2	1	2	5	Fair
Esteban(1998)	3	1	2	6	Good
Jin (1998)	3	1	2	6	Good
Martin (1998)	2	1	2	5	Fair
Lee (2000)	3	2	2	7	Good
Chia (2001)	3	1	2	6	Good
Sato (2002)	2	1	2	5	Fair
Toyoda (2004)	2	1	2	5	Fair
Tsukuma (2006)	3	1	3	7	Good
Chen (2006)	2	1	2	5	Fair
Yaghi (2006)	3	1	3	7	Good
Chen (2007)	2	1	2	5	Fair
Jung (2007)	2	1	3	6	Good
Changchien (2008)	2	2	2	6	Good
Lim (2009)	2	2	2	6	Good
Redaniel (2009)	2	2	2	6	Good
Tanaka (2009)	2	1	3	6	Good
Laudico (2010)	2	1	2	5	Fair
Law (2011)	4	1	3	8	Good
Kudo (2011)	2	1	2	5	Fair
Sumitsawan (2011)	3	1	3	7	Good
Matsuda (2011)	2	1	2	5	Fair
Redaniel (2011)	2	2	2	6	Good
Chen (2011)	3	1	3	7	Good
Chia (2011)	3	1	3	7	Good
Jayalekshmi (2011)	2	1	3	6	Good
Jayant (2011)	2	1	3	6	Good
Martin (2011)	2	1	2	5	Fair
Sankaranarayanan (2011)	2	1	2	5	Fair
Jung (2011)	2	1	2	5	Fair
Sriplung (2011)	2	1	2	5	Fair
Xiang (2011)	4	1	3	8	Good
Xishan (2011)	4	1	3	8	Good
Jung (2012)	2	1	2	5	Fair
Azmawati (2012)	2	2	2	6	Good
Chen (2013)	2	1	3	6	Good
Jung (2013)	2	1	2	5	Fair
Ito (2013)	2	1	2	5	Fair
Norsa'adah (2013)	2	1	2	5	Fair
Pinheiro (2014)	2	2	2	6	Good
Ito (2014)	2	1	2	5	Fair
Jung (2014)	2	1	2	5	Fair
Fan (2014)	3	2	3	8	Good
Somboon (2014)	2	2	2	6	Good
Jung (2015)	2	1	2	5	Fair
Maringe (2015)	2	2	2	6	Good
Zeng (2015)	3	2	2	7	Good
Liu (2015)	2	2	2	6	Good
Xiao (2015)	2	2	3	7	Good
Zhang (2015)	2	1	2	5	Fair
Zheng (2015)	2	1	2	5	Fair
Zhu (2015)	2	1	2	5	Fair
Chiang (2016)	2	1	2	5	Fair

EXCLI Journal 2020;19:108-130 – ISSN 1611-2156
Received: September 23, 2019, accepted: November 19, 2019, published: January 13, 2020

Author (year)	Selection	Comparability	Outcome	Total	Quality
Kudo (2016)	2	1	2	5	Fair
Oh (2016)	2	1	2	5	Fair
Nakagawa-Senda (2017)	2	1	2	5	Fair
Jung (2017)	2	1	2	5	Fair
Li (2017)	2	1	2	5	Fair
Chen (2018)	2	1	2	5	Fair
Chien (2018)	1	1	3	5	Fair
Jung (2018)	2	1	2	5	Fair
Li-Hsin (2018)	2	2	2	6	Good

Supplementary Appendix 3: Result of meta-regression for one (**A**), three (**B**) and five-year (**C**) survival rate of liver cancer based on year of study

(A)

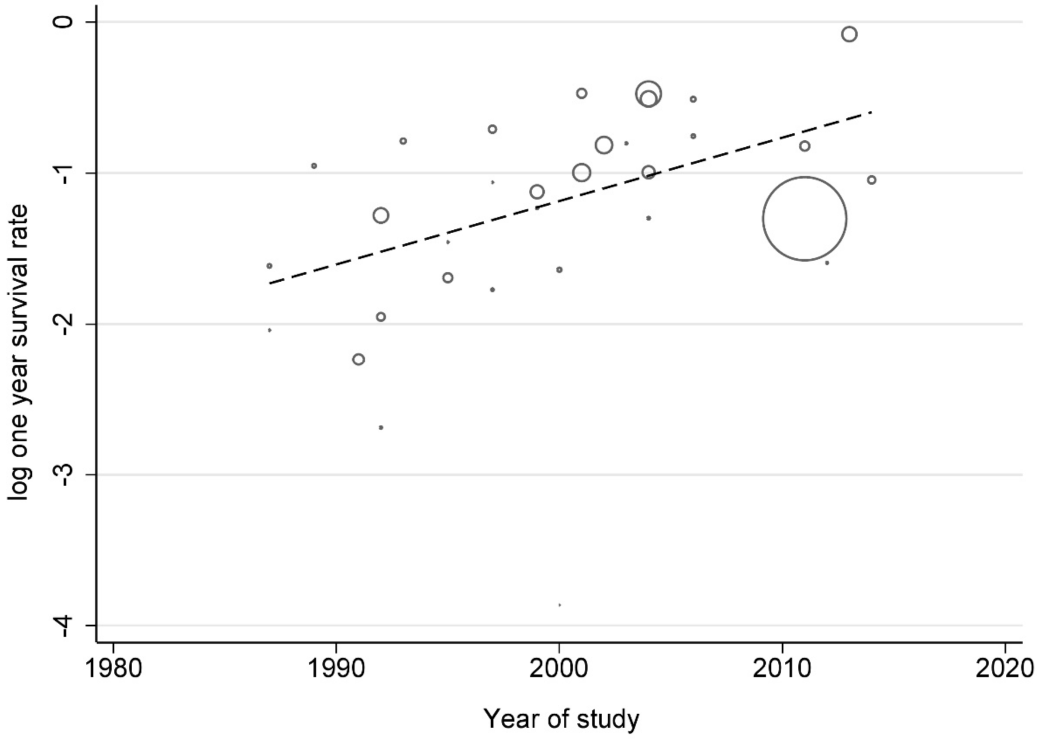

EXCLI Journal 2020;19:108-130 – ISSN 1611-2156
Received: September 23, 2019, accepted: November 19, 2019, published: January 13, 2020

(B)

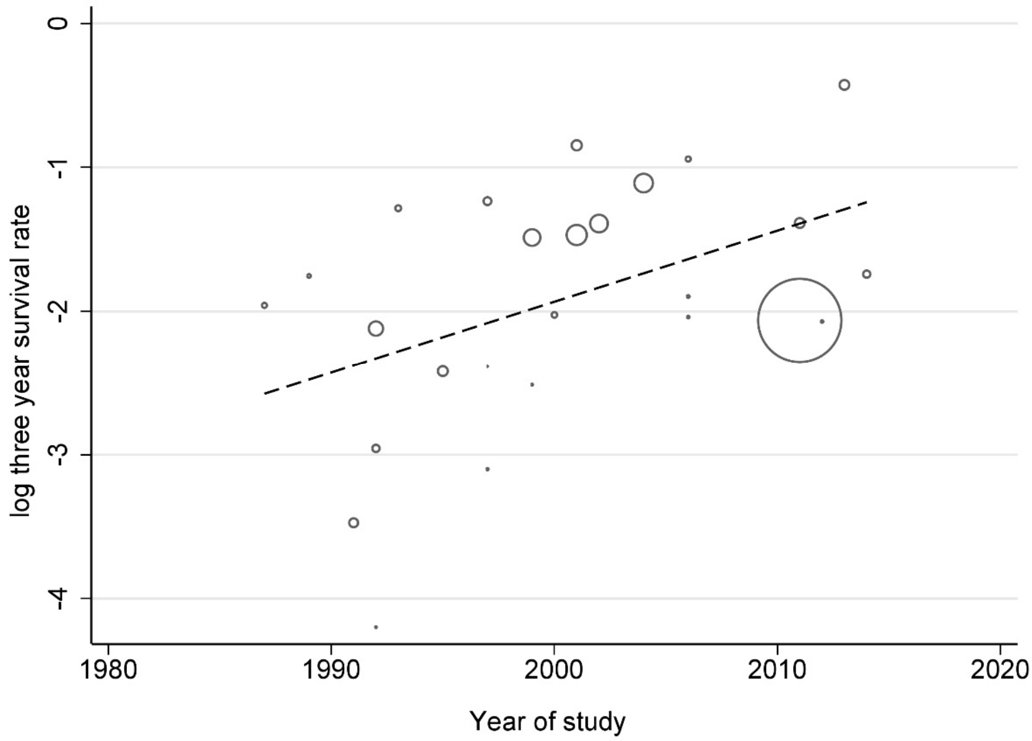

(C)

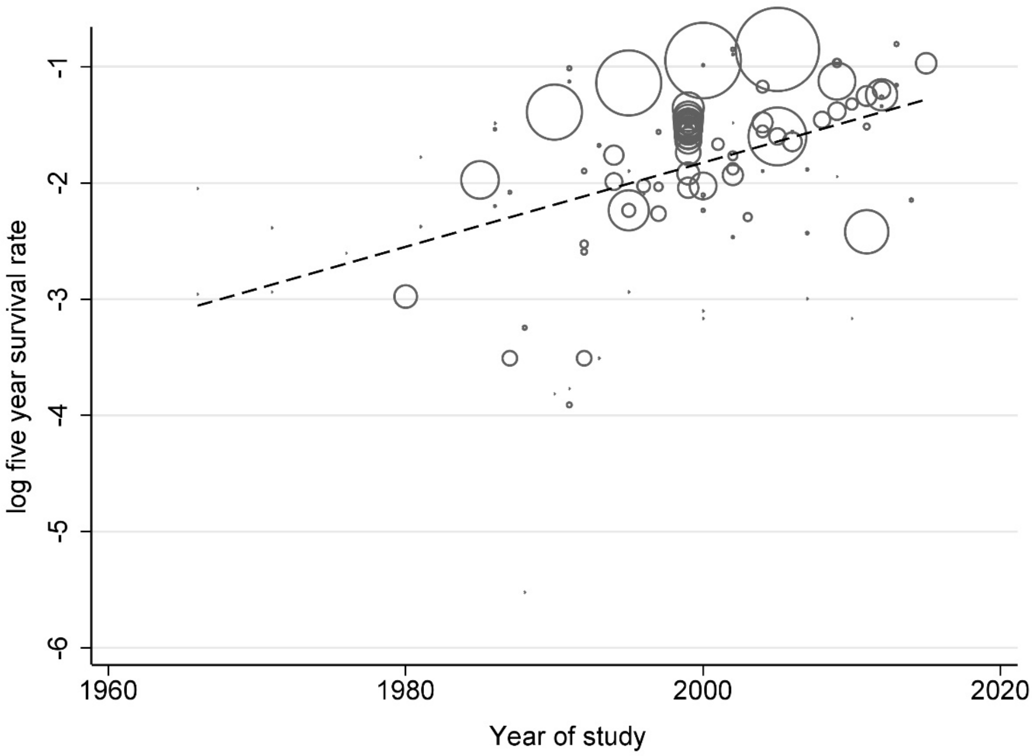

Supplement: Supplementary material [file EXCLI-19-108-s-001.pdf]
